# The Clinical Trials Directive: How Is It Affecting Europe's Noncommercial Research?

**DOI:** 10.1371/journal.pctr.0010013

**Published:** 2006-06-30

**Authors:** Markus Hartmann, Florence Hartmann-Vareilles

In this article, we examine and discuss the current situation for noncommercial clinical trials in Europe—two years after a new legal framework entered into force. The Clinical Trials Directive, issued in 2001 [1], sought to regulate clinical research in a uniform way across Europe. The basic aims underpinning its development were to cut red tape, speed up research and development, enhance the quality of investigational drugs, harmonise procedures, increase the transparency of the clinical research process, and last, but not least, enforce patient protection. The Directive required that trialists and sponsors ensure ethical review and authorisation by competent national authorities before enrolling participants, drug manufacture in line with Good Manufacturing Practice guidelines, and rigorous observance of the Good Clinical Practice (GCP) principles during the conduct of the trial. Furthermore, the Directive required that any changes related to the execution of the clinical study, and its final results, be reported to the supervising authorities. To transpose the Directive into national law, each European Union (EU) member state has had to change its established legal framework for clinical drug research to meet the requirements of the Directive.

Since that time, the Directive has fundamentally changed the face of clinical research in Europe. While the pharmaceutical industry has become accustomed to intervening early in political decisionmaking and legislative processes, public and academic research institutions have taken more time to develop a common action plan in response to the Directive [2,3]. Perhaps due to the legal complexity of the subject, responses to the Directive's impact on noncommercial research have been limited to surveys [4,5]. Attempts to convey the current situation in the EU are restricted by language and information barriers and by insufficient resources for conducting a Europe-wide analysis.

## Harmonisation Still Outstanding

**Figure 1:**
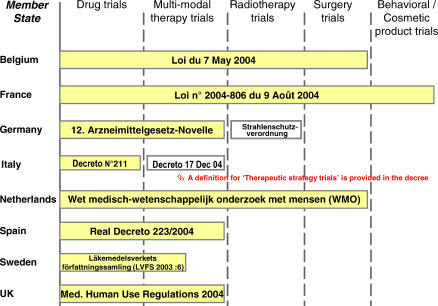
Schematic Presentation of Differences in the Scope of National Laws Governing Clinical Trials in Europe Legislative acts in each member state were recently revised to cope with the requirements of the CTD 2001/20/EC. Shadowed boxes indicate revised legislation in response to the Directive. The German ordinance on irradiation protection (Strahlenschutzverordnung) and the Italian decree on clinical research for the improvement of clinical practice (Decreto 17 Dec 04) constitute two accompanying legal acts. The term *multi-modal therapy trials* refers to trials that evaluate the effects of drug therapy together with other forms of medical intervention, e.g., irradiation, surgery, other procedures.

When trying to understand how the EU Clinical Trials Directive (CTD 2001/20) has been applied across Europe, our first question is whether the original goal of harmonisation has been achieved. A comparison of revised legislative texts in eight EU member states shows that differences remain for many types of clinical trials (Figure 1). France appears to have the most comprehensive legal definition on biomedical research, with public authorities rigorously supervising all kind of trials, including those on cosmetics. The Directive was set up with the intention of governing clinical research into medicinal products (medicines, drugs). Therefore, problems may arise in applying the Directive to trials that investigate complex modes of treatment (e.g., surgical techniques, or combinations of drugs and devices). As shown in Figure 1, many borderline situations will occur in future trials, making it difficult to determine whether a trial falls under the new regulation or not. Such problems are even more far-reaching if a multicentre trial investigating complex modes of treatment is carried out in many different European countries.

There is the same lack of harmonisation in relation to the way the Directive applies to noncommercial trials across the EU. The Directive itself contains only two areas where exceptions are allowed for noncommercial trials, but these only apply where the drug is already marketed and used in line with its marketing authorisation: 1) labelling requirements for the investigational drug are less stringent; 2) dossier submission to national authorities can be relaxed in certain ways.

These two exceptions have been adopted in similar ways throughout Europe, but they apply only to a small subset of trials, known as *therapeutic use studies*.

The term *noncommercial trial* emerged from the Clinical Trial Directive. But only two of the eight member states compared in Figure 1 (Italy and Belgium) have put in place legislation that recognises the potential benefits of noncommercial clinical trials to patients and public health. Other member states claim that such exceptions are difficult to reconcile with the universally protective character of the legislation on medical research in humans, in view of the repeated mentions of universal patient protection in the GCP-Directive 2005/28/EC [6], issued as an addendum to the CTD 2001/20. In addition, no common definition exists in the EU to explain what a noncommercial trial is. Such trials may be very wide-ranging, including experimental research into new, unapproved chemical or biological entities, and therapeutic use research into established, approved drugs. For example, therapeutic use research examines the effects of varying dosage or application schedules, with the intention of improving day-to-day medical practice.

## Major Obstacles and Unsolved Issues

Since the Directive's application, key problems have been reported by academic researchers in published letters and conference presentations [2,3]: 1) a requirement for single sponsorship for multicentre and, more demandingly, pan-European multicentre studies; 2) definition of the investigational medicinal product (IMP). The key question here is what portion of a patient's comprehensive medication scheme constitutes background and/or supportive medication and what portion is exerting the pharmacological effect under investigation; 3) free-of-charge supply principle of the investigated medicine, which requires that trial sponsors provide the IMP for free; 4) increased cost of insurance coverage; 5) increased cost of quality assurance systems for supervision of ongoing trials; and 6) increased cost of submissions to ethics committees, national authorities, and fees for GCP inspections, carried out by national authorities or the European Medicines Agency (EMEA).

These are a particular burden for noncommercial trials, and for investigators who want to start their own, independent research.

A comparative analysis of member states' provisions illustrates that the promises of the Directive have not been fulfilled (Table 1). To resolve liability issues, in each country cascades of agreements have been required between investigators and hospitals, hospitals and their public or private shareholders, and between hospitals' owners and the state authorities. It appears that member states with tax-financed public health systems, such as in the UK or Sweden, have found it easier to solve the liability problem of public sponsors. For example, in the UK, the Medicines and Healthcare Products Regulatory Agency (MHRA), Universities UK, and the Department of Health have carried out a regulatory impact assessment [7]. This assessment has enabled charities and associations in the UK to act as a sponsor in multinational studies taking place across the EU.

**Table 1:**
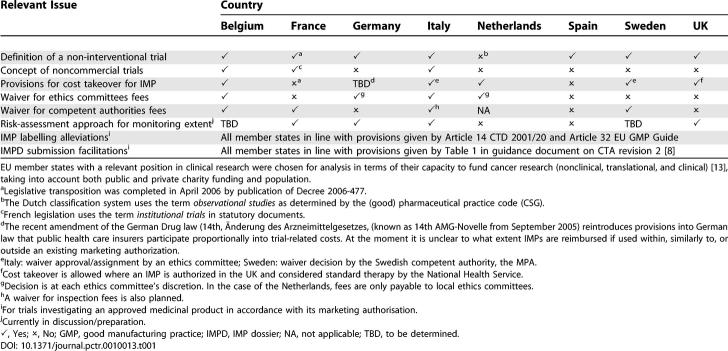
Country-Specific Provisions for Noncommercial Clinical Trials in Different EU Member States

The European Commission has recently provided a new definition for non-IMPs (NIMP), clarifying the current situation considerably [8]. The new definition states that “support or escape medication for preventive, diagnostic or therapeutic reasons” or medications “used in accordance with the protocol to induce a physiological response” should be distinguished from an IMP.

However, many other aspects of the Directive still remain to be commonly understood or adopted (Table 1). For example, the issue of reimbursement of commercially available drug supplies by public health insurance systems is still problematic. For trials in HIV or oncology that test the best therapeutic use of innovative medicines in combination with existing therapy schemes, the free-of-charge supply implicates a cost takeover of investigated medicines as well as of established co-medications. In Italy, Belgium, Sweden, and the Netherlands, specific mechanisms have been set up for noncommercial trials, which allow for public cost takeover for tested drugs and co-medications (support or background treatments). Although the processes differ between countries, these provisions nevertheless show the willingness of some national policymakers to encourage patient-oriented research and access to new therapies. In the fields of pediatrics and research into rare diseases, clinical trials provide a key role in giving patients access to innovative treatments.

At a glance, it can be seen that the problems caused by the CTD 2001/20 arise from the multitude of liabilities effected by each clinical trial. Many of the open questions have to be solved by each individual EU member state. Each state is responsible for tort and liability issues, for public health care provisions including reimbursement, and for science and research [9]. Therefore, rules for noncommercial trials in the EU continue to be divergent: on the one hand, clinical trials are subject to the harmonisation obligations of the EU, and, on the other hand, they are subject to the legal codes of each individual member state where the trial is conducted. This discrepancy inevitably leads to a significant rise in administrative workload for large, multinational clinical trials because such trials must obey a host of different administrative requirements in each country in which they are conducted. Today, sponsors of multinational trials have to cope with the need to obey complex and differing codes of law across all the countries they are working in. In most EU member states, the laws have several enclosures in the form of lengthy guidance documents with dozens of chapters and up to 100 text pages.

## Beneficial Effects

Patient protection was highlighted as one of the main reasons behind the Directive. One controversial issue that remains relates to the protection of incapable, critically ill patients [10,11]. The Directive requires that surrogate consent from a nominated legal representative is provided before critically ill patients without relatives can be enrolled in a clinical trial. This requirement has been tackled in different ways by each member state, and has an obvious impact on emergency care research.

Historically, investigational medicines used in trials could be produced in facilities without extensive Good Manufacturing Practice. Now, the Directive lays out strict regulations governing manufacture of investigational drugs (technical rules and guidelines set out in Volume 4 of the EudraLex catalogue [12]). The Directive therefore minimises risks for trial participants that might arise from defective or poor quality investigational drugs.

The Directive's request to provide a financing or fundraising plan enhances transparency. The obligatory registration of clinical trials and the intended publication of key information on ongoing trials in public databases (such as the planned public module of the EudraCT database) constitute a major beneficial achievement. Increased transparency is also a key part of the revised EU pharmaceutical legislation, which has been in place since November 2005 [9]. This legislation aims to provide the public with better information on clinical trials, medicines, and public health issues.

The Directive might also be seen as a route towards reducing the number of unoriginal or poorly conducted clinical trials (i.e., protocols not followed properly), or those that are poorly managed (i.e., trial supervision is inadequate or data management procedures not well done or biased). The Directive may lead to better quality of research in both commercial and noncommercial fields with the hope that in the future the number of “me-too” trials and products might decline. In this context, the limited funding for clinical research in the EU will play a major role. Governmental and nongovernmental funding bodies will need high-calibre expertise if they want to base future funding decisions on project excellence.

## Prospects

More than ever before, funding will be a key issue for noncommercial clinical research. Remarkable work in this respect has been done recently by the European Cancer Research Managers Forum, which has recently examined Europe-wide funding in cancer research [13]. Based on figures obtained before the EU was enlarged, the level of funding for noncommercial clinical research in the US is five times higher per capita than in Europe. Europe's capacity to preserve its expertise and leading position in clinical research therefore crucially depends on the available public and private funding. Funding needs to be increased if academic institutions are to maintain their role in supporting research that will improve patient care.

At present, it's not clear how the problems reported in this article can be resolved. A new Directive (GCP-Directive 2005/28/EC [6]), was due to be implemented through the EU by the end of January 2006. This new Directive gives member states more flexibility to legislate on noncommercial trials, stating that “the conditions under which the noncommercial research is conducted by public researchers and the places where this research takes place, make the application of certain of the details of good clinical practice unnecessary or guaranteed by other means.” But will countries who have just made changes to legislation, and who voted for a rather restrictive handling of noncommercial clinical research, reopen the gates for more and easier research? Any answer is speculative, particularly given that the EU Commission's eagerly awaited, additional guidance on noncommercial trials has not yet been released.

The European approach to regulation of clinical trials is an administrative and procedural one. A drug's sponsorship and development stages only play subordinated roles. For drugs with an existing marketing authorisation, the Summary of Product Characteristics serves as a landmark for all future trials.

As regulations become more and more burdensome, many therapeutic interventions are excluded from routine practice, restricted to off-label application, or used in clinical trials only.

In the past, clinical trials carried out with paediatric patients or with those with rare diseases often legitimized the use of innovative medicines. Results from such trials were often necessary to convince health insurers to take over the costs of therapy for drugs not yet accepted for official reimbursement. Recent forecasts and first survey results gave evidence for a 30%–50% drop in the number of new noncommercial clinical trials started in the EU since 2004 [14]. This leads to fewer opportunities to gain access to innovative drugs. In paediatric oncology, there are fears that the decline of clinical research will result in poorer outcomes for patients.

**Figure 2:**
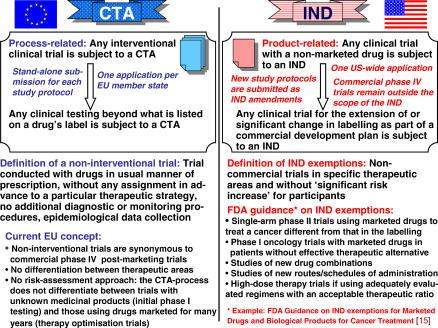
Comparison of the EU CTA and the US IND Application Procedures For noncommercial, patient-focused research, supplemental guidelines were issued in the US, whereas in the EU, exemptions from the clinical trial application (CTA) process and applicable GCP principles are restricted to post-authorisation safety studies. Observational studies using epidemiological methods are generally exempted from regulation in the EU and US.

How can the situation be improved? In the EU, any systematic change in a drug's application from the approved use described on its label means that any clinical investigation must conform to all requirements of the CTD 2001/20. In many member states, policies for graduated application review or risk assessment strategies for clinical trials do not exist, although these are urgently needed. A comparison with the US is illuminating (Figure 2) [15,16]. In the US, a single initial investigational new drug (IND) dossier has to be established for a nonmarketed drug before clinical tests can start. All subsequent trial protocols are submitted as amendments to the IND. In contrast, the EU Directive requires a stand-alone dossier submission for each trial protocol, and in each member state. The US Food and Drug Administration (FDA) allows exemptions from IND submission for non-interventional trials and trials for patients with life-threatening diseases or a lack of good therapeutic alternatives. This offers clinicians more choice in patient-focused research. Establishing such a risk-assessment approach along with a single European trial evaluation and approval process for multinational clinical studies should be top priorities for policymakers in order to keep the European clinical trials' environment competitive [17].

## Abbreviations

CTA: clinical trial application
CTD 2001/20: Clinical Trials Directive
EU: European Union
GCP: Good Clinical Practice
IMP: investigational medicinal product
IND: investigational new drug
